# COVID-19 Induced Environments, Health-Related Quality of Life Outcomes and Problematic Behaviors: Evidence from Children with Syndromic Autism Spectrum Disorders

**DOI:** 10.1007/s10803-022-05619-7

**Published:** 2022-06-07

**Authors:** Corneliu Bolbocean, Kayla B Rhidenour, Maria McCormack, Bernhard Suter, J Lloyd Holder

**Affiliations:** 1 Nuffield Department of Primary Care Health Sciences, University of Oxford, Oxford, UK; 2 Department of Communication, Baylor University, Waco, TX USA; 3 Jan and Dan Duncan Neurological Research Institute, Texas Children’s Hospital, 1250 Moursund St. Suite 925, Houston, TX 77030 USA; 4 Division of Neurology and Developmental Neuroscience, Department of Pediatrics, Baylor College of Medicine, Houston, TX USA

**Keywords:** COVID-19, Autism spectrum disorders, HRQoL, Phelan–McDermid syndrome, Rett Syndrome, SYNGAP1-ID

## Abstract

Between July 2020 and January 2021, 230 principal caregivers completed a questionnaire to measure proxy-assessed health-related quality of life outcomes (HRQoL), behavioral outcomes in children with syndromic autism spectrum disorders and COVID-19 induced changes to lifestyle and environments. HRQoL and behavioral outcomes reported earlier during the pandemic were generally worse compared to those reported later. COVID-19 induced reduction to a caregiver’s mental health appointments, and hours spent watching TV were associated with decreases in HRQoL and increased the likelihood of problematic behaviors. Increasing time outdoors and time away from digital devices were positively associated with HRQoL and behaviors and might protect children from COVID-19 induced restrictions.

## Introduction

The COVID-19 pandemic disrupted the daily lives of children and their caregivers. Recent literature links the COVID-19 pandemic with high rates of adverse outcomes experienced by children (Liu et al., 2020; Singh et al., 2020) particularly from those in vulnerable groups and their families. Specifically, results have shown that the COVID-19 pandemic is associated with an increased risk of negative mental health and neurodevelopmental outcomes among children diagnosed with neurodevelopmental disorders (Masi et al., 2021; Summers et al., 2021; Ehrler et al., 2021; Courtenay and Perera 2020). Overall, the evidence is not fully conclusive. Initial studies have generally highlighted the adverse impact of the COVID-19 pandemic on children and adolescents while other contradictory evidence found either no impact, or in some cases, positive effects of the pandemic (Bruining et al., 2021; Achterberg et al., 2021; Koenig et al., 2021).

Preliminary evidence suggests that COVID-19 has adversely impacted individuals diagnosed with Autism Spectrum Disorders (ASDs). Children diagnosed with ASD are known to be particularly vulnerable due to their difficulty with adapting to new situations such as COVID-19 induced lockdowns (Pellicano et al., 2021; den Houting 2020; Pellicano and Stears 2020; Eshraghi et al., 2020). Research has found that COVID-19 negatively impacted ASD children’s mental and physical status (Lugo-Marín et al., 2021; Colizzi et al., 2020) as well as self-regulation and co-operation skills (Morris et al., 2021).

However, despite the existing evidence documenting the impact on health outcomes of COVID-19 on children diagnosed with ASD, there is no research to date that has ascertained the impact on health-related quality of life outcomes (HRQoL) in this pediatric population. Moreover, it is currently unknown if and how environmental changes due to COVID-19 are associated with HRQoL outcomes in ASD children. Multi-dimensional HRQoL measures provide a comprehensive, holistic approach to evaluate the consequences of the COVID-19 pandemic. HRQoL measures move beyond the standard clinical effectiveness framework, and are able to deliver the meaningful data required for comprehensive health economics and policy evaluations for Drugs et al. (2006), Chim et al. (2010), Excellence (2013) and are also used in clinical settings Mouillet et al. (2021), Nguyen et al. (2021), Tian-hui et al. (2005), and Pais-Ribeiro (2004).

This study had the following aims: (a) to ascertain HRQoL outcomes using valid and reliable measures during COVID-19 for children diagnosed with three genetic disorders which strongly predispose a child to syndromic ASD: Phelan–McDermid syndrome (PMD), Rett Syndrome (RTT) and *SYNGAP1*-related intellectual disability (*SYNGAP1*-ID) and (b) to determine the relationship between the pandemic-induced lifestyle changes, HRQoL, behavior problems and the mechanisms around which environmental changes can impact behaviors. Finally, (c) we compared children’s outcomes during earlier stages of COVID-19 with later phases of the outbreak. We chose these populations because these disorders strongly predispose to a diagnosis of autism (Holder Jr et al., 2019; Vlaskamp et al., 2019; Jimenez-Gomez et al., 2019; Phelan et al., 2018; De Rubeis et al., 2018; Oberman et al., 2015; Berryer et al., 2013; Neul 2012; Kaufmann et al., 2012) and since these neurodevelopmental disorders display complex associations with autistic features and behavior problems consistent with ASD Holder Jr et al. (2019); Phelan et al. (2018); Kaufmann et al. (2012). Genetic syndromes with high prevalence of autism are often referred to as syndromic autism because of their comorbid phenotypes of intellectual disability, epilepsy and dysmorphic features (Benvenuto et al., 2009; Sztainberg and Zoghbi 2016). Moreover, because these disorders have defined genetic etiologies, each potentially has a more homogeneous phenotype than idiopathic autism.

PMD is characterized by early global developmental delay that leads to intellectual disability in nearly 100% of affected individuals (De Rubeis et al., 2018). The most severely impacted developmental domain is language, and this, in combination with social behavior deficits as well as sensory processing abnormalities, frequently leads to a diagnosis of autism. Approximately 35% of individuals with PMD also develop epilepsy. Individuals with *SYNGAP1*-ID similarly typically present with global developmental delay that leads to a diagnosis of intellectual disability (Holder Jr et al., 2019). In contrast to PMD, over 90% of individuals affected by *SYNGAP1*-ID develop epilepsy, often intractable. However, similar to PMD, children with *SYNGAP1*-ID frequently also have social and sensory processing deficits which in combination with severe abnormalities in language development lead to a diagnosis of autism. For RTT, early development of affected girls can be normal with sudden regression of developmental skills at 1–4 years of age (Neul, 2012). This includes loss of both social skills as well as language again leading to an autism diagnosis. Uniquely for RTT, prominent loss of purposeful hand use is common.

We hypothesized that positive changes to children’s pandemic induced environments had a positive relationship on children’s HRQoL and behavioral outcomes across each diagnosis. Furthermore, we tested the hypothesis that an earlier phase of the pandemic was associated with worse outcomes in children compared to later stages of the COVID-19 pandemic. We also aimed to test the hypothesis that pandemic-induced healthcare constraints and negative changes to children’s environments had adversely influenced children’s HRQoL and behavioral outcomes for children with PMD, RTT and *SYNGAP1*-ID.

## Methods

### Study Background and Sample

For this study, 230 families with children diagnosed with PMD, RTT and *SYNGAP1*-ID completed self-report questionnaires in order to ascertain COVID-19 induced lifestyle changes or changes in their environments between July 2020 and January 2021. The principal caregivers completed a questionnaire for proxy-assessed health status in children. Study participants were distributed as follows: 138 (60%) of the participants were diagnosed with PMD, 50 (22%) with *SYNGAP1*-ID and 42 with RTT (18%). The study was approved by the Baylor College of Medicine’s Institutional Review Board. A recruitment email provided access to an online Qualtrics survey through the following organizations: Phelan–McDermid Syndrome Foundation, RettSyndrome.org, *SYNGAP1* Foundation and SynGAP Research Fund, Inc. Participants had to read and understand English as well as have access to the Internet in order to complete the web-based questionnaire. All respondents were required to give their informed consent online prior to completing the questionnaire.

### Outcome Variables: HRQoL Variables and Problematic Behaviors

Our main dependent variables were children’s HRQoL and behavioral outcomes. HRQoL of the study’s participants was ascertained using the $\hbox {PedQL}^{TM}$ Version 4.0 Generic Core Scales self-report questionnaire. This instrument was developed to measure the main dimensions of health as well as role functioning (school/day care) Varni et al. (1999). The proxy (third person) version of the questionnaire contains 23 items examining physical functioning (8 items), emotional functioning (5 items), social functioning (5 items) and school functioning (5 items). Each item utilized a 5-point response scale (0 = never a problem; 1 = almost never a problem; 2 = sometimes a problem; 3 = often a problem; 4 = almost always a problem). Items were reverse-scored and linearly transformed to a 0-100 scale (0 = 100, 1 = 75, 2 = 50, 3 = 25, 4 = 0). Thus, higher scores indicate better HRQoL. For scale and total scores, the mean was computed as the sum across all items divided by the number of items answered. Furthermore, a psychosocial health summary score was calculated as the mean score over the number of items answered across the emotional, social, and school functioning scales. We considered the following behavioral outcomes: indicator variables which denoted that the child startled easily sometimes or often; that the child had angry outburst sometimes or often and if the child seemed in a daze sometimes or often during the COVID-19 outbreak.^1^

### COVID-19 Exposures

We designed a survey to investigate the association between the COVID-19 outbreak and the following domains: overall health, behaviors, school activities, access to healthcare, daily routine, as well as, basic household and medical expenses. Specific exposures of interest were indicator variables for: COVID-19 reduced caregiver’s mental health appointments; COVID-19 reduced access to medical health care services; during COVID-19 some money was left over; the child spent more than 2 hours outdoor each day during the COVID-19 outbreak; the child had access to a yard or other outdoor space; the child had in-person, phone or video contact at least once per week; on average during the COVID-19 pandemic the child spent at least 3 hours per day playing computer games; on average since the COVID-19 pandemic the child spent at least 3 hours per day watching TV; the child has been somewhat or very successful in completing the recommended homework during COVID-19. Furthermore, we used an interaction variable between time outdoors during COVID-19 of more than 2 hours each day and annual family income over $\$55$ thousand (U.S. dollars) per year.

### Covariates

Covariates included in the multivariable models were informed by previous theoretical or empirical models (Olson et al., 2021; Thomas et al., 2012; Larsson et al., 2005) and included: age at assessment (years), English as the main language (yes (referent), no), caregiver’s highest educational qualification, marital status (married (referent), not married), race (white (referent), not white), and income.

### Empirical Analyses

Differences in characteristics between children with PMD, *SYNGAP1*-ID and RTT were tested using the ANOVA test for continuous variables and Chi-squared test for categorical variables. ANOVA and Chi-squared tests had also been used to assess differences by diagnosis status related to COVID-19 variables and HRQoL outcomes. Differences in $\hbox {PedQL}^{TM}$ scale, psychosocial and total scores, between children with PMD, *SYNGAP1*-ID and RTT were estimated using an ANOVA test under standard distributional assumptions. Finally, we performed separate multivariable regressions to explore the adjusted association between COVID-19 related factors on HRQoL measured using $\hbox {PedQL}^{TM}$ and clinically relevant outcomes. Furthermore, to test for the presence of indirect socio-economic effects moderating the association between time spent outdoors and children’s outcomes we examined the association of interaction variable between time outdoors during COVID-19 of 2 or more hours each day and annual family income over $\$55$ thousand U.S. dollars per year. Fixed effects multivariate regression analyses were performed where the type of diagnosis was modeled as fixed effects due to condition-specific behavioral effects by diagnosis, which corrected for the fixed unobserved heterogeneity within each diagnosis (PMD, *SYNGAP1*-ID and RTT). We used the Ordinary Least Squares (OLS) estimator to perform the multivariable regressions. Linear probability models were fitted to investigate the adjusted association between COVID-19 induced covariates and the probability of developing problematic behaviors.

The data was collected between July 2020 and January 2021, and the response rate was not uniformly distributed across each day. However, the median participation rate was achieved on September 25, 2020. For the purpose of our study, we defined the early COVID-19 phase as the time between the start of data collection up to and including September 25, 2020, and the late COVID-19 phase after September 25, 2020 up to January 2021. We employed a 1 to 1 random matching (Rothman, 2012; Stuart, 2010) to compare children’s outcomes during earlier and later phases of the outbreak across all diagnoses.

Furthermore, we estimated the impact of the later COVID-19 phase by randomly matching upon the clinical diagnosis as this type of matching has been shown as an effective method to control for clinical and behavioral profiles of disorders (Rothman, 2012; Stuart, 2010). Differences between the time before or on September 25, 2020, and after this date matched observations were ascertained using the Student t-test for unequal variances. However, due to small sample sizes this analysis was restricted for PMD children only. Differences between early and late groups were ascertained using the Student t-test for unequal variances. Confidence intervals (CI) were computed using bias-corrected and accelerated bootstrap method (BCa). Statistical analyses were conducted using Stata 16.0 (Stata Corp, College Station, TX). P-values of 0.05 or less were considered statistically significant.

## Results

### Baseline Characteristics

Table 1 reports baseline characteristics of the study’s population across each diagnosis. Of the 230 participants there were no differences found across diagnoses (138 with PMD, 50 with *SYNGAP1*-ID and 42 with RTT) among the following variables: child’s age, age at diagnosis, participation in early behavioral intervention, primary language spoken at home, caregiver’s marital status, race or ethnicity, caregiver’s relationship to the child, and participation in the Special Supplemental Nutrition Program for Women, Infants, and Children (WIC) program.^2^ However, statistically significant differences were recorded across diagnoses within the following domains: the child’s gender (*p*-value < 0.01) as expected because RTT mostly impacts girls, type of health insurance (*p*-value <0.01), and the estimated annual household income (*p*-value < 0.01). The overall evidence suggested that the majority of socioeconomic covariates were largely balanced across diagnoses.

**Table 1:** Demographic Characteristics by Type of Genetic Diagnosis

	PMD	SYNGAP1-ID	RTT	Total	*p*-value
n (%)	138 (60.0)	50 (21.7)	42 (18.3)	230 (100.0)	
*Child’s age in years*, mean (sd)	11.05 (5.71)	11.78 (5.02)	12.30 (4.57)	11.48 (5.33)	0.49
*Age at diagnosis in years*, mean (sd)	9.44 (4.47)	8.42 (4.76)	9.56 (4.54)	9.15 (4.55)	0.55
*Gender*, n (%)					
Female	46 (46.9)	24 (51.1)	29 (96.7)	99 (56.6)	
Male	52 (53.1)	23 (48.9)	1 (3.3)	76 (43.4)	0.00
*Child received any type of early behavioral intervention*, n (%)					
No	46 (46.5)	21 (44.7)	14 (48.3)	81 (46.3)	
Yes	53 (53.5)	26 (55.3)	15 (51.7)	94 (53.7)	0.95
*Child’s COVID-19 status*, n (%)					
No	94 (96.9)	42 (97.7)	27 (93.1)	163 (96.4)	
Yes	3 (3.1)	1 (2.3)	2 (6.9)	6 (3.6)	0.55
*Child’s Health during COVID-19* , n (%)					
Average	11 (12.1)	8 (19.0)	5 (19.2)	24 (15.1)	
Excellent	37 (40.7)	14 (33.3)	9 (34.6)	60 (37.7)	
Good	42 (46.2)	20 (47.6)	12 (46.2)	74 (46.5)	
Terrible	1 (1.1)	0 (0.0)	0 (0.0)	1 (0.6)	0.87
*Primary language spoken in home*, n (%)					
English	78 (77.2)	38 (80.9)	34 (91.9)	150 (81.1)	
Other	18 (17.8)	7 (14.9)	2 (5.4)	27 (14.6)	
Spanish	5 (5.0)	2 (4.3)	1 (2.7)	8 (4.3)	0.42
*Estimated total annual household income*, n (%)					
Less than or equal to $34999	27 (27.6)	8 (17.0)	5 (13.9)	40 (22.1)	
$35000–$74999	23 (23.5)	10 (21.3)	16 (44.4)	49 (27.1)	
$$\ge$$ $75000	48 (49.0)	29 (61.7)	15 (41.7)	92 (50.8)	0.04
*Health insurance status?*, n (%)					
Medicaid AND Private	34 (34.3)	16 (34.0)	13 (36.1)	63 (34.6)	
Medicaid and/or Medicare ONLY	15 (15.2)	7 (14.9)	12 (33.3)	34 (18.7)	
No health insurance	15 (15.2)	1 (2.1)	0 (0.0)	16 (8.8)	0.01
*Participation in Women Infant Children program*, n (%)					
No	79 (79.8)	40 (85.1)	24 (66.7)	143 (78.6)	
Yes	20 (20.2))	7 (14.9)	12 (33.3)	39 (21.4)	0.12
*Caregiver’s marital status*, n (%)					
Divorced	5 (5.0)	6 (12.8)	4 (10.8)	15 (8.2)	
Living with Partner	7 (7.0)	3 (6.4)	1 (2.7)	11 (6.0)	
Married	83 (83.0)	37 (78.7)	31 (83.8)	151 (82.1)	
Never Married	3 (3.0)	1 (2.1)	0 (0.0)	4 (2.2)	
Separated	2 (2.0)	0 (0.0)	1 (2.7)	3 (1.6)	0.65
*Caregiver’s race/ethnicity*, n (%)					
American Indian or Alaska Native	1 (1.0)	0 (0.0)	0 (0.0)	1 (0.5)	
Asian	4 (4.0)	2 (4.3)	3 (8.3)	9 (4.9)	
Black or African American	1 (1.0)	2 (4.3)	0 (0.0)	3 (1.6)	
Other	6 (5.9)	2 (4.3)	1 (2.8)	9 (4.9)	
White	89 (88.1)	41 (87.2)	32 (88.9)	162 (88.0)	0.72
*Caregiver’s level of education*, n (%)					
< High School	11 (11.0)	3 (6.4)	3 (8.1)	17 (9.2)	
College Degree	38 (38.0)	15 (31.9)	13 (35.1)	66 (35.9)	
Graduate or Professional Degree	37 (37.0)	23 (48.9)	12 (32.4)	72 (39.1)	
High School or GED equivalent	8 (8.0)	0 (0.0)	7 (18.9)	15 (8.2)	
Technical School	6 (6.0)	6 (12.8)	2 (5.4)	14 (7.6)	0.08

For child’s age and age at diagnosis *p*-value is based on an ANOVA test. For other variables *p*-value is based on a Chi-squared test. WIC stands for The Special Supplemental Nutrition Program for Women, Infants, and Children

### COVID-19 Induced Environments & Problematic Behaviors

Tables 2 and 3 show the distribution of COVID-19 related covariates across each diagnosis. Statistically significant differences were detected across the diagnoses within multiple questions which ascertained the impact of COVID-19 on lifestyle changes and problematic behaviors in children. Results showed statistical significance in relation to a child’s behavior’ when we asked if the child startled easily, had angry outburst, or seemed to be in a daze more often during the pandemic. For example, almost 90% of *SYNGAP1*-ID parents reported angry outbursts sometimes or often versus the other disorders where it was around 60%. Also, over 45% of *SYNGAP1*-ID parents reported their child startled easily, sometimes or often compared to PMD and RTT around 35%. Furthermore, almost 75% of *SYNGAP1*-ID parents reported their child seemed in a daze sometimes or often, followed by RTT around 66% and PMD 44%.

Additionally, we found statistical significance in a child’s behavior with regard to their time spent outdoors with 35% of individuals with RTT rarely going outside compared with 7% and 14% of *SYNGAP1*-ID and PMD respectively (Table 3). Moreover, we also found significance across genetic diagnoses in variables dealing with caregiver and family support, measuring whether or not a child had expressed fears that they, a parent or another close family member could contact and/or die from COVID-19.

**Table 2:** Child’s Problematic Behaviors during COVID-19 Outbreak by Type of Genetic Diagnosis

	PMD	SYNGAP1-ID	RTT	Total	*p*-value
n (%)	138 (60.0)	50 (21.7)	42 (18.3)	230 (100.0)	
*Since becoming aware of the COVID-19 outbreak, how often has your child*					
Seemed happy and satisfied with his/her life, n (%)					
Not at all	1 (1.2)	0 (0.0)	0 (0.0)	1 (0.7)	
Often	58 (69.9)	26 (70.3)	19 (67.9)	103 (69.6)	
Sometimes	24 (28.9)	11 (29.7)	9 (32.1)	44 (29.7)	0.93
Had difficulty sleeping, n (%)					
Not at all	30 (33.3)	9 (20.9)	5 (17.9)	44 (27.3)	
Often	28 (31.1)	16 (37.2)	7 (25.0)	51 (31.7)	
Sometimes	32 (35.6)	18 (41.9)	16 (57.1)	66 (41.0)	0.20
Startled easily, n (%)					
Not at all	50 (63.3)	21 (53.8)	18 (64.3)	89 (61.0)	
Often	16 (20.3)	2 (5.1)	8 (28.6)	26 (17.8)	
Sometimes	13 (16.5)	16 (41.0)	2 (7.1)	31 (21.2)	0.00
Had angry outburst, n (%)					
Not at all	36 (40.9)	3 (7.0)	11 (40.7)	50 (31.6)	
Often	27 (30.7)	14 (32.6)	5 (18.5)	46 (29.1)	
Sometimes	25 (28.4)	26 (60.5)	11 (40.7)	62 (39.2)	0.00
Seemed to have a sense of time slowing down, n (%)					
Not at all	32 (60.4)	9 (36.0)	10 (66.7)	51 (54.8)	
Often	11 (20.8)	8 (32.0)	2 (13.3)	21 (22.6)	
Sometimes	10 (18.9)	8 (32.0)	3 (20.0)	21 (22.6)	0.26
Seemed in a daze, n (%)					
Not at all	43 (53.8)	9 (25.7)	8 (33.3)	60 (43.2)	
Often	9 (11.3)	2 (5.7)	4 (16.7)	15 (10.8)	
Sometimes	28 (35.0)	24 (68.6)	12 (50.0)	64 (46.0)	0.01
Seemed to try to avoid thoughts and feelings about COVID-19, n (%)					
Not at all	26 (76.5)	9 (69.2)	2 (66.7)	37 (74.0)	
Often	2 (5.9)	0 (0.0)	0 (0.0)	2 (4.0)	
Sometimes	6 (17.6)	4 (30.8)	1 (33.3)	11 (22.0)	0.74
Had distressing dreams, n (%)					
Not at all	30 (73.2)	10 (58.8)	2 (28.6)	42 (64.6)	
Often	4 (9.8)	1 (5.9)	2 (28.6)	7 (10.8)	
Sometimes	7 (17.1)	6 (35.3)	3 (42.9)	16 (24.6)	0.13
Been distressed when he/she sees something that reminds him/her of COVID-19, n (%)					
Not at all	33 (78.6)	10 (62.5)	5 (83.3)	48 (75.0)	
Often	4 (9.5)	2 (12.5)	0 (0.0)	6 (9.4)	
Sometimes	5 (11.9)	4 (25.0)	1 (16.7)	10 (15.6)	0.65
Did things that he/she had outgrown or acted younger than current age? n (%)					
Not at all	44 (60.3)	20 (54.1)	13 (68.4)	77 (59.7)	
Often	14 (19.2)	3 (8.1)	3 (15.8)	20 (15.5)	
Sometimes	15 (20.5)	14 (37.8)	3 (15.8)	32 (24.8)	0.19
*How successful has your child been in completing the recommended homeschool activities?* n (%)					
Not at all successful	28 (37.3)	12 (30.8)	6 (24.0)	46 (33.1)	
Prefer not to answer	8 (10.7)	2 (5.1)	1 (4.0)	11 (7.9)	
Somewhat successful	27 (36.0)	21 (53.8)	14 (56.0)	62 (44.6)	
Very successful	12 (16.0)	4 (10.3)	4 (16.0)	20 (14.4)	0.42
* How stressful have these homeschool activities been for your child?* n (%)					
Not at all stressful	18 (26.5)	5 (13.2)	6 (23.1)	29 (22.0)	
Prefer not to answer	5 (7.4)	1 (2.6)	1 (3.8)	7 (5.3)	
Somewhat stressful	24 (35.3)	22 (57.9)	12 (46.2)	58 (43.9)	
Very stressful	21 (30.9)	10 (26.3)	7 (26.9)	38 (28.8)	0.40

*p*-value is based on a Chi-squared test

**Table 3:** Child’s Environments during COVID-19 Outbreak by Type of Genetic Diagnosis

	PMD	SYNGAP1-ID	RTT	Total	*p*-value
n (%)	138 (60.0)	50 (21.7)	42 (18.3)	230 (100.0)	
*How many hours per day does your child sit and watch TV or videos?* n (%)					
1 hour	8 (9.9)	6 (14.6)	4 (14.3)	18 (12.0)	
2 hours	15 (18.5)	4 (9.8)	3 (10.7)	22 (14.7)	
3 hours	11 (13.6)	9 (22.0)	2 (7.1)	22 (14.7)	
4 hours	5 (6.2)	5 (12.2)	3 (10.7)	13 (8.7)	
5 hours or more	22 (27.2)	10 (24.4)	12 (42.9)	44 (29.3)	
Less than 1 hour	14 (17.3)	5 (12.2)	2 (7.1)	21 (14.0)	
My child does not watch TV or videos	6 (7.4)	2 (4.9)	2 (7.1)	10 (6.7)	0.57
*How many hours per day does your child use a computer or play computer?* n (%)					
1 hour	8 (9.9)	3 (7.0)	3 (10.7)	14 (9.2)	
2 hours	10 (12.3)	4 (9.3)	2 (7.1)	16 (10.5)	
3 hours	7 (8.6)	5 (11.6)	1 (3.6)	13 (8.6)	
4 hours	5 (6.2)	5 (11.6)	0 (0.0)	10 (6.6)	
5 hours or more	17 (21.0)	15 (34.9)	9 (32.1)	41 (27.0)	
Less than 1 hour	11 (13.6)	6 (14.0)	5 (17.9)	22 (14.5)	
My child does not have screen time on any devices	22 (27.2)	5 (11.6)	8 (28.6)	35 (23.0)	
Prefer not to answer	1 (1.2)	0 (0.0)	0 (0.0)	1 (0.7)	0.55
*How much in-person/phone/video contact has your child had with friends?* n (%)					
Don’t know	10 (12.5)	5 (11.6)	4 (14.3)	19 (12.6)	
Every few days	25 (31.3)	11 (25.6)	8 (28.6)	44 (29.1)	
Once per week	30 (37.5)	21 (48.8)	11 (39.3)	62 (41.1)	
Prefer not to answer	5 (6.3)	2 (4.7)	0 (0.0)	7 (4.6)	
Several times per day	10 (12.5)	4 (9.3)	5 (17.9)	19 (12.6)	0.85
*Does your child have access to a yard or other outdoor space where you live?* n (%)					
No	7 (8.6)	2 (4.7)	4 (14.3)	13 (8.6)	
Yes	74 (91.4)	41 (95.3)	24 (85.7)	139 (91.4)	0.37
*How much time has your child spent outdoors each day during the COVID-19?* n (%)					
1 hour or less	17 (21.0)	11 (25.6)	10 (35.7)	38 (25.0)	
1–2 hours	25 (30.9)	15 (34.9)	5 (17.9)	45 (29.6)	
2–3 hours	16 (19.8)	10 (23.3)	3 (10.7)	29 (19.1)	
>4 hours	12 (14.8)	4 (9.3)	0 (0.0)	16 (10.5)	
Rarely went out	11 (13.6)	3 (7.0)	10 (35.7)	24 (15.8)	0.02
*How much stress have you/your family experienced of potentially being exposed to COVID-19* n (%)					
Don’t know	0 (0.0)	1 (2.3)	1 (3.6)	2 (1.3)	
Not at all stressed	22 (27.2)	3 (7.0)	3 (10.7)	28 (18.4)	
Somewhat stressed	32 (39.5)	23 (53.5)	10 (35.7)	65 (42.8)	
Very stressed	27 (33.3)	16 (37.2)	14 (50.0)	57 (37.5)	0.04
*How have your family finances worked out at the end of the month?* n (%)					
Just enough to make ends meet	24 (30.0)	18 (41.9)	13 (46.4)	55 (36.4)	
Not enough to make ends meet	7 (8.8)	2 (4.7)	4 (14.3)	13 (8.6)	
Prefer not to answer	2 (2.5)	3 (7.0)	0 (0.0)	5 (3.3)	
Some money left over	47 (58.8)	20 (46.5)	11 (39.3)	78 (51.7)	0.20
*How much the COVID-19 has changed your access to medical healthcare? * n (%)					
Mild	24 (29.6)	19 (44.2)	9 (32.1)	52 (34.2)	
Moderate. Delays or cancellations in appointments.	28 (34.6)	13 (30.2)	10 (35.7)	51 (33.6)	
No change	27 (33.3)	6 (14.0)	8 (28.6)	41 (27.0)	
Severe. Unable to access care resulting in moderate/severe impact on health.	2 (2.5)	5 (11.6)	1 (3.6)	8 (5.3)	0.10
*How much the COVID-19 outbreak has changed your access to mental health?* n (%)					
Mild	19 (25.7)	15 (38.5)	8 (30.8)	42 (30.2)	
Moderate. Delays or cancellations in appointments.	12 (16.2)	7 (17.9)	2 (7.7)	21 (15.1)	
No change	36 (48.6)	13 (33.3)	13 (50.0)	62 (44.6)	
Severe. Unable to access care resulting in moderate/severe impact on health.	7 (9.5)	4 (10.3)	3 (11.5)	14 (10.1)	0.64

*p*-value is based on a Chi-squared test

### HRQoL

Table 4 presents descriptive statistics for the PedsQL scale, psychosocial and total scores across PMD, *SYNGAP1*-ID and RTT. The evidence demonstrated that there were no significant differences across the mean scores for school functioning (PMD highest 57.12, followed by RTT (48.80) and *SYNGAP1*-ID 45.68). However, results showed that mean scores were significantly different for physical functioning (PMD 45.68, *SYNGAP1*-ID 41.50 followed by RTT 19.95, *p*-value < 0.01), emotional functioning (PMD 71.22, *SYNGAP1*-ID 62.03 followed by RTT 61.20, *p*-value <0.05), psychosocial functioning (PMD 60.65, RTT 53.00 followed by *SYNGAP1*-ID 51.71, *p*-value <0.05) and for the overall score (PMD 55.78, *SYNGAP1*-ID 48.20 followed by RTT 41.67, *p*-value <0.01).

**Table 4:** PedsQL Generic Core Scales Health-related Quality of Life Scores by Type of Genetic Diagnosis

	PMD	SYNGAP1-ID	RTT	Total	*p*-value
n (%)	138 (60.0)	50 (21.7)	42 (18.3)	230 (100.0)	
Physical functioning, mean (sd)	45.68 (27.22)	41.50 (23.75)	19.95 (16.68)	39.94 (26.28)	0.00
Emotional functioning, mean (sd)	71.22 (23.62)	62.03 (16.85)	61.20 (19.43)	66.76 (21.52)	0.03
Social functioning, mean (sd)	53.38 (24.00)	46.86 (24.37)	50.40 (15.74)	50.93 (22.95)	0.33
School functioning, mean (sd)	57.12 (28.73)	45.68 (22.56)	48.80 (27.77)	52.37 (27.25)	0.07
Psychosocial functioning, mean (sd)	60.65 (20.51)	51.71 (15.84)	53.00 (17.21)	56.68 (19.05)	0.02
Total Score, mean (sd)	55.78 (20.88)	48.20 (16.33)	41.67 (14.42)	51.10 (19.31)	0.00

*p*-value is based on a ANOVA test

### The Association Between COVID-19 Induced Lifestyle Changes, Children’s HRQoL and Problematic Behaviors

Table 5 separately reports results from two models: one which assessed the adjusted association of COVID-19 induced lifestyle changes and pre-specified clinical and sociodemographic covariates on total PedsQL score, and the other model which ascertained the association of COVID-19 factors on psychosocial PedsQL scores only. We found that COVID-19 induced constraints related to caregivers’ mental health appointments was associated with a decrement of 14.5 points (95% CI − 27.26, − 1.82) in PedsQL total score and a decrement of 21.25 (95% CI − 35.76, − 7.25) in PedsQL psychosocial score. Empirical models identified the adjusted negative effect of a child watching at least 3 hours of TV per day on his/her HRQoL which was associated with a decrement of 13.76 in PedsQL total score (95% CI − 21.41, − 6.11) and a decrement of 12.88 (95% CI − 20.21, − 5.55) for PedsQL psychosocial score. However, if caregivers reported their child spent more than two hours outdoors per day, there was a positive association with PedsQL total score of 6.63 (95% CI − 0.69, 13.95) adjusted for all other covariates. Furthermore, we found that marital status and level of education were statistically significant predictors of PedsQL during the COVID-19 outbreak. We note here that other variables, while not statistically significant, were of the plausible direction.

**Table 5:** Multivariable Analyses of proxy-assessed HRQoL Outcomes (OLS regression)

Outcome	Covariates	$\beta$	se($\beta$)	Lower 95% CI	Upper 95% CI	*p*-value
PedsQL Total	RTT	$-\,$ 7.169	5.491	$-\,$ 18.083	3.745	0.192
	*SYNGAP1*-ID	$-\,$ 4.955	3.706	$-\,$ 12.321	2.411	0.181
	MH Appointment	$-\,$ 14.539	6.400	$-\,$ 27.261	$-\,$ 1.818	0.023
	Access Health	$-\,$ 6.807	7.927	$-\,$ 22.562	8.948	0.390
	Finance	$-\,$ 0.589	3.251	$-\,$ 7.051	5.872	0.856
	Time Outdoor	6.632	3.682	$-\,$ 0.687	13.950	0.072
	Income and Time Outdoor	6.020	3.171	$-\,$ 0.261	12.291	0.062
	Yard	$-\,$ 0.445	6.849	$-\,$ 14.059	13.169	0.948
	Human Contact	4.564	3.369	$-\,$ 2.131	11.260	0.175
	Computer Intensity	2.147	3.854	$-\,$ 5.512	9.807	0.577
	TV Intensity	$-\,$ 13.761	3.850	$-\,$ 21.414	$-\,$ 6.108	0.000
	Homeschool	$-\,$ 0.406	4.268	$-\,$ 8.890	8.078	0.924
PedsQL Psychosocial	RTT	$-\,$ 2.918	5.626	$-\,$ 14.101	8.265	0.604
	*SYNGAP1*-ID	$-\,$ 6.142	3.760	$-\,$ 13.616	1.331	0.102
	MH Appointment	$-\,$ 21.253	7.045	$-\,$ 35.256	$-\,$ 7.249	0.003
	Access Health	$-\,$ 2.502	8.319	$-\,$ 19.037	14.032	0.764
	Finance	$-\,$ 0.288	3.484	$-\,$ 7.213	6.637	0.934
	Time Outdoor	3.571	3.634	$-\,$ 3.652	10.794	0.326
	Income and Time Outdoor	2.641	3.092	$-\,$ 3.471	8.753	0.390
	Yard	$-\,$ 3.109	7.373	$-\,$ 17.763	11.545	0.673
	Human Contact	2.253	3.477	$-\,$ 4.657	9.164	0.517
	Computer Intensity	0.656	3.630	$-\,$ 6.559	7.872	0.857
	TV Intensity	$-\,$ 12.880	3.688	$-\,$ 20.211	$-\,$ 5.549	0.000
	Homeschool	$-\,$ 2.094	4.147	$-\,$ 10.337	6.148	0.614

*Notes:* MH Appointment indicates COVID-19 reduced caregiver’s mental health appointments. Access Health indicates COVID-19 reduced access medical health care services. Finance indicates if during COVID-19 some money were left over. Time Outdoor indicates if child spent outdoors each day during COVID-19 more than 2 hours each day. Yard indicates if the child has access to a yard or other outdoor space. Human Contact indicates that a child had at least once per week in-person, phone or video contact. Computer Intensity indicates that on average since the COVID-19 the child spent playing computer games at least 3 hours per day. TV Intensity indicates that on average since the COVID-19 the child spent watching TV at least 3 hours per day. Homeschool indicates if child been somewhat or very successful in completing the recommended homework during COVID-19. Income and Time Outdoor variable is an interaction between time outdoors during COVID-19 more than 2 hours each day and annual family income over $\$55$ thousand per year was significant across all models. Models adjusted for demographic controls such as: caregiver’s marital status, education, race and income. Robust standard errors reported

Table 6 reports results from OLS models which assessed the association between COVID-19 factors and problematic behaviors. There was a positive association between a higher reported reduction in a caregiver’s mental health appointments due to COVID-19 and all three negative child outcomes (startled easily, angry outburst and daze). Moreover, time spent outdoors was found to decrease the risk of angry outbursts and the child being in a daze. These two findings are consistent with previous results reported in Table 5 which explored the association between COVID-19 induced lifestyle changes on HRQoL outcomes. Furthermore, the evidence showed that the interaction variable between spending more than 2 hours outdoors during COVID-19 and annual family income over $\$55$ thousand U.S. dollars per year was a statistical predictor for several variables. In particular, this interaction variable had a virtually identical relationship with the indicator for a child easily startling and had the indicator for a child’s angry outburst around − 0.19 (95% CI − 0.35, − 0.02).

**Table 6:** Multivariable Analyses of Problematic Behaviors (OLS regression)

Outcome	Covariates	$\beta$	se($\beta$)	Lower 95% CI	Upper 95% CI	*p*-value
Startled Easily	RTT	$-\,$ 0.330	0.165	$-\,$ 0.659	$-\,$ 0.001	0.046
	*SYNGAP1*-ID	$-\,$ 0.096	0.103	$-\,$ 0.301	0.110	0.354
	MH Appointment	0.644	0.210	0.226	1.062	0.002
	Access Health	0.272	0.251	$-\,$ 0.228	0.772	0.279
	Finance	$-\,$ 0.202	0.108	$-\,$ 0.417	0.013	0.062
	Time Outdoor	$-\,$ 0.110	0.111	$-\,$ 0.331	0.110	0.318
	Income and Time Outdoor	$-\,$ 0.192	0.094	$-\,$ 0.35.	$-\,$ 0.024	0.033
	Yard	$-\,$ 0.132	0.259	$-\,$ 0.647	0.384	0.611
	Human Contact	0.028	0.092	$-\,$ 0.154	0.210	0.758
	Computer Intensity	0.056	0.122	$-\,$ 0.186	0.298	0.646
	TV Intensity	0.057	0.120	$-\,$ 0.183	0.297	0.635
	Homeschool	$-\,$ 0.008	0.118	$-\,$ 0.243	0.228	0.949
Angry Outburst	RTT	0.046	0.175	$-\,$ 0.303	0.395	0.794
	*SYNGAP1*-ID	0.242	0.083	0.078	0.406	0.003
	MH Appointment	0.382	0.132	0.120	0.644	0.004
	Access Health	$-\,$ 0.141	0.135	$-\,$ 0.409	0.127	0.296
	Finance	$-\,$ 0.193	0.089	$-\,$ 0.370	$-\,$ 0.017	0.029
	Time Outdoor	$-\,$ 0.191	0.092	$-\,$ 0.375	$-\,$ 0.008	0.038
	Income and Time Outdoor	$-\,$ 0.182	0.080	$-\,$ 0.340	$-\,$ 0.021	0.032
	Yard	0.035	0.177	$-\,$ 0.317	0.387	0.845
	Human Contact	$-\,$ 0.007	0.086	$-\,$ 0.177	0.164	0.937
	Computer Intensity	$-\,$ 0.023	0.109	$-\,$ 0.241	0.194	0.830
	TV Intensity	0.069	0.109	$-\,$ 0.148	0.286	0.527
	Homeschool	$-\,$ 0.025	0.112	$-\,$ 0.248	0.198	0.825
Daze	RTT	0.372	0.177	0.019	0.724	0.035
	*SYNGAP1*-ID	0.198	0.116	$-\,$ 0.033	0.430	0.088
	MH Appointment	0.619	0.222	0.175	1.062	0.005
	Access Health	$-\,$ 0.150	0.261	$-\,$ 0.669	0.370	0.565
	Finance	0.010	0.108	$-\,$ 0.205	0.225	0.928
	Time Outdoor	$-\,$ 0.300	0.114	$-\,$ 0.527	$-\,$ 0.073	0.008
	Income and Time Outdoor	$-\,$ 0.053	0.091	$-\,$ 0.230	0.131	0.582
	Yard	0.655	0.200	0.257	1.054	0.001
	Human Contact	$-\,$ 0.014	0.102	$-\,$ 0.217	0.189	0.890
	Computer Intensity	0.066	0.132	$-\,$ 0.197	0.329	0.618
	TV Intensity	0.175	0.133	$-\,$ 0.091	0.441	0.190
	Homeschool	$-\,$ 0.110	0.127	$-\,$ 0.364	0.144	0.389

Startled easily indicates that the child startled easily sometimes or often. Angry Outburst indicates that the child had angry outburst sometimes or often. Daze is an indicator if child seemed in a daze sometimes or often during COVID-19 outbreak

### Early vs Late COVID-19 on HRQoL and Problematic Behaviors

Table 7 reports results from comparing HRQoL outcomes and problematic behaviors by date of responses. This study started data collection in June, 2020; however, on September 25th, 2020, a little over half of respondents (55.7%) had completed the survey. The overall evidence in Table 7 shows that generally PedsQL scores were higher for the late phase vs early phase. Additionally, psychosocial functioning scores were 5.97 CI (95% CI − 0.22, 12.15) higher for respondents during the later phase. Furthermore, the proportion of reported problematic behaviors decreased slightly by − 0.14 (95% CI − 0.30, 0.02) for children who startled easily sometimes or often and by − 0.16 (95% CI − 0.32, 0.01) for children who seemed in a daze sometimes or often during COVID-19. Interestingly, when we repeated the analysis using September 5th as a cutoff date (as around 20% of the responses were received before this date) the associations provided greater contrast. Thus, psychosocial functioning scores were 7.71 CI (95% CI 0.71, 14.71) higher for respondents after September 5th and statistically significant at 5% and total PedsQL scores were 6.19 (95% CI − 0.94, 13.32). We also found statistically significant decrements in the proportion of reports related to angry outburst − 0.31 (95% CI − 0.48, − 0.15) and being in a daze − 0.26 (95% CI − 0.46, − 0.07). However, these analyses did not adjust for clinical diagnosis, and thus, for heterogeneity across the compared groups.

**Table 7:** HRQoL and Problematic Behaviors by Early vs Late COVID-19

	Before September 25, 2020	After September 25, 2020	Difference	SD	Lower 95% CI	Upper 95% CI	*p*-value
n (%)	128 (55.7)	102 (44.3)					
Physical Functioning, mean (95% CI)	39.64 (34.27; 45.01)	40.32 (33.52; 47.12)	0.68	4.36	$-\,$ 7.94	9.30	0.88
Emotional Functioning, mean (95% CI)	63.80 (59.24; 68.36)	70.40 (65.13; 75.66)	6.60	3.54	$-\,$ 0.39	13.60	0.06
Social Functioning, mean (95% CI)	48.50 (43.40; 53.60)	53.92 (48.48; 59.37)	5.42	3.82	$-\,$ 2.13	12.97	0.16
School Functioning, mean (95% CI)	49.17 (43.79; 54.55)	56.31 (49.02; 63.59)	7.14	4.53	$-\,$ 1.81	16.08	0.11
Psychosocial Functioning, mean (95% CI)	54.00 (50.07; 57.93)	59.97 (55.18; 64.76)	5.97	3.13	$-\,$ 0.22	12.15	0.06
Total, mean (95% CI)	49.38 (45.44; 53.32)	53.24 (48.29; 58.19)	3.86	3.19	$-\,$ 2.44	10.16	0.23
Startled Easily, mean (95% CI)	0.45 (0.34; 0.56)	0.31 (0.20; 0.43)	$-\,$ 0.14	0.08	$-\,$ 0.30	0.02	0.09
Angry Outburst, mean (95% CI)	0.73 (0.63; 0.82)	0.63 (0.51; 0.74)	$-\,$ 0.10	0.07	$-\,$ 0.25	0.05	0.19
Daze, mean (95% CI)	0.64 (0.53; 0.75)	0.48 (0.36; 0.61)	$-\,$ 0.16	0.08	$-\,$ 0.32	0.01	0.06
	Before September 5, 2020	After September 5, 2020					
n (%)	47 (20.4)	183 (79.6)					
Physical Functioning, mean (95% CI)	37.99 (30.96; 45.02)	40.62 (35.45; 45.78)	2.62	4.96	$-\,$ 7.17	12.42	0.60
Emotional Functioning, mean (95% CI)	61.25 (56.18; 66.32)	68.68 (64.38; 72.98)	7.43	4.02	$-\,$ 0.52	15.38	0.06
Social Functioning, mean (95% CI)	46.58 (39.14; 54.02)	52.48 (48.18; 56.78)	5.90	4.32	$-\,$ 2.64	14.44	0.17
School Functioning, mean (95% CI)	44.43 (37.44; 51.41)	55.09 (49.72; 60.47)	10.67	5.13	0.52	20.81	0.04
Psychosocial Functioning, mean (95% CI)	50.96 (46.27; 55.66)	58.67 (54.92; 62.43)	7.71	3.54	0.71	14.71	0.03
Total, mean (95% CI)	46.50 (41.87; 51.13)	52.69 (48.86; 56.52)	6.19	3.61	$-\,$ 0.94	13.32	0.09
Startled Easily, mean (95% CI)	0.45 (0.34; 0.56)	0.31 (0.20; 0.43)	$-\,$ 0.07	0.10	$-\,$ 0.26	0.12	0.49
Angry Outburst, mean (95% CI)	0.73 (0.63; 0.82)	0.63 (0.51; 0.74)	$-\,$ 0.31	0.08	$-\,$ 0.48	$-\,$ 0.15	0.00
Daze, mean (95% CI)	0.64 (0.53; 0.75)	0.48 (0.36; 0.61)	$-\,$ 0.26	0.10	$-\,$ 0.46	$-\,$ 0.07	0.01

Startled easily indicates that the child startled easily sometimes or often. Angry Outburst indicates that the child had angry outburst sometimes or often. Daze is an indicator if child seemed in a daze sometimes or often during COVID-19 outbreak. *p*-value is based on Student t-test for unequal variances

To control for heterogeneity across clinical profiles we randomly matched observations before and after September 25th, upon clinical diagnosis.^3^ Given the sample size, we were only able to match PMD patients. Table 8 reports results from comparing early vs late COVID-19 HRQoL and problematic behaviors using a sample of 65 matched pairs with PMD. The evidence shows that total PedsQL scores were 8.14 higher (95% CI − 1.33, 17.62) for PMD respondents after September 25, 2020. Similarly, emotional functioning scores were 11.61 (95% CI 0.86, 22.37) higher but statistically significant at 5%, for PMD participants after September 25, 2020 and physical functioning score were 11.57 higher (95% CI − 0.52, 23.97) after the cutoff date. Furthermore, we also found statistically significant decrements in the proportion of reports that a child startled easily − 0.27 (95% CI − 0.49, − 0.05) among PMD respondents September 25, 2020.

**Table 8:** HRQoL and Problematic Behaviors by Early vs Late COVID-19: Matched Analysis for PMD Patients

	Before September 25	After September 25	Difference	SD	Lower 95% CI	Upper 95% CI	*p*-value
n (%)	65 (50.0)	65 (50.0)					
Physical Functioning, mean (95% CI)	39.99 (31.02; 48.97)	51.57 (43.33; 59.81)	11.57	6.17	− 0.52	23.67	0.06
Emotional Functioning, mean (95% CI)	64.85 (56.17; 73.53)	76.46 (70.04; 82.89)	11.61	5.40	0.86	22.37	0.03
Social Functioning, mean (95% CI)	52.19 (44.19; 60.18)	55.61 (47.91; 63.31)	3.42	5.72	− 7.99	14.84	0.55
School Functioning, mean (95% CI)	53.33 (45.05; 61.62)	61.22 (51.79; 70.65)	7.88	6.46	− 4.77	20.55	0.22
Psychosocial Functioning, mean (95% CI)	56.99 (50.20; 63.79)	64.43 (58.27; 70.59)	8.14	4.79	− 1.25	17.54	0.09
Total, mean (95% CI)	51.87 (44.99; 58.75)	60.02 (53.73; 66.30)	8.14	4.75	− 1.33	17.62	0.09
Startled Easily, mean (95% CI)	0.51 (0.35; 0.68)	0.24 (0.10; 0.38)	− 0.27	0.11	− 0.49	− 0.05	0.01
Angry Outburst, mean (95% CI)	0.59 (0.43; 0.75)	0.61 (0.47; 0.76)	0.02	0.11	− 0.19	0.24	0.83
Daze, mean (95% CI)	0.54 (0.38; 0.71)	0.39 (0.24; 0.54)	− 0.15	0.11	− 0.38	0.08	0.18

Startled easily indicates that the child startled easily sometimes or often. Angry Outburst indicates that the child had angry outburst sometimes or often. Daze is an indicator if child seemed in a daze sometimes or often during COVID-19 outbreak. *p*-value is based on Student t-test for unequal variances

## Discussion

This is the first study to provide evidence regarding the association between COVID-19 induced lifestyle changes, HRQoL and problematic behaviors in children diagnosed with syndromic autism. The exposure-outcome relationship was directly observed and the data structure enabled us to estimate the association between COVID-19 induced lifestyle changes on syndromic autistic children’s HRQoL and behaviors by using multivariate regression models and matching models. We documented HRQoL measured using $\hbox {PedQL}^{TM}$ and clinically relevant health outcomes in children diagnosed with syndromic autism (PMD, *SYNGAP1*-ID and RTT) along with the environment they experienced during the early phases of the COVID-19 pandemic. Furthermore, we estimated the association between COVID-19 induced factors and HRQoL with problematic behaviors using rich socioeconomically data. The data structure enabled us to identify the relationship between COVID-19 induced factors and HRQoL outcomes, as well as, clinically significant outcomes and correct for condition-specific unobserved heterogeneity by using fixed effects estimators. Furthermore, our study was able to contrast HRQoL outcomes during earlier and later phases of the outbreak using matching methods.

Our study focused on behavior problems and the mechanisms around which environmental changes can impact the behaviors of children with syndromic ASD. The overall evidence in this study highlights that COVID-19 changes to lifestyles and environments primarily had negative consequences for HRQoL and problematic behaviors in syndromic autistic children. Specifically, the results indicated that COVID-19 reduced a caregiver’s mental health appointments, and the hours a child spent watching TV were associated with significant decreases in $\hbox {PedQL}^{TM}$ scores and with probability increase in problematic behaviors. On the other hand, there are positive impacts for HRQoL found in our results as well. Hours spent outdoors had a positive relationship on HRQoL among children with PMD, *SYNGAP1*-ID and RTT and probability decrease in problematic behaviors. These findings demonstrate that COVID-19 induced factors had a consistent relationship across different measures utilized to measure children’s outcomes during the pandemic. In other words, negative changes to lifestyles were consistently shown to be associated with negative outcomes.

Our results showed how children’s outcomes may be exacerbated through caregiving stress during COVID-19 as the pandemic induced reduction of a caregiver’s mental health appointments were negatively associated with HRQoL in syndromic ASD children. This is consistent with findings from the literature which links parental mental health and outcomes in children (Kamis, 2021; Pierce et al., 2020), however, as far as we are aware, the parental access to mental health care services was not previously directly linked to HRQoL in children during COVID-19. This is likely a fruitful area of further investigation. Moreover, our results imply that the cost-effectiveness analysis of novel interventions designed to ensure that access to health services continues during possible outbreaks should consider changes to children’s HRQoL.

Interestingly, we also found evidence to suggest that socio-economic effects might moderate the association between time spent outdoors and children’s outcomes as the interaction variable between time outdoors during COVID-19 of more than 2 hours each day and annual family income over $\$55$ thousand U.S. dollars per year was significant across several models. However, possible mediating effects of socio-economic factors should be explored in future research. Furthermore, the evidence led us to conclude that the distribution of PedsQL scores is different across PMD, *SYNGAP1*-ID and RTT during the pandemic which is consistent with a previously reported finding Bolbocean et al. (2021). At the same time, HRQoL scores among PMD are higher compared to the other two genetic conditions which has also been previously observed.

Results demonstrated that children’s outcomes started to improve gradually at the end of the third quarter and leading into the fourth quarter of 2020. The strongest evidence is provided by matched analysis of PMD patients whom experienced improvements in HRQoL and clinically relevant behaviors. This is likely due to adjustments to new COVID-19 environments or to possible relaxation of pandemic-imposed restrictions. Future research should disentangle these two effects across early and later stages of the epidemic.

Methods employed and the findings from our study might provide an avenue to reconcile the seemingly conflictive evidence regarding the overall impact of COVID-19 on children’s outcomes. In particular, this study demonstrates that HRQoL and behavioral outcomes reported earlier during the pandemic were generally worse compared to those reported later. This implies that studies assessing the impact of COVID-19 on children’s outcomes might benefit from controlling for pandemic specific phases such as early vs late COVID-19 stages during data collection. From a clinical perspective, controlled trials prospectively investigating either providing mental health appointments for parents of children with syndromic autism or promoting time spent outdoors for these children warrant consideration based upon our findings.

### Strengths and Limitations

The major strength of this study is the timely collection of rich and novel data during COVID-19 which ascertained exposure-outcome relationships consistently across genetic disorders that strongly predispose children to syndromic ASD. Furthermore, we used different measures, HRQoL and observed behaviors, to assess the relationship between COVID-19 induced environments and children’s outcomes. Thus, we used validated and reliable HRQoL measures which are holistic constructs (Barile et al., 2013; Moriarty et al., 2003), that are highly correlated with widely used health metrics including morbidity, mortality, and healthcare and provide data required for comprehensive health economics, and policy evaluations for Drugs et al. (2006), Chim et al. (2010), and Excellence (2013). Furthermore, a high number of subjects with rare diseases is also a strength as the combined diagnosis matched sample of 230 individuals of HRQoL data during the COVID-19 pandemic.

We acknowledge the limitations of our findings. First, the present study used a cross-sectional design as the data on family and children’s environments were only collected during the pandemic. Also, the sample of the study might not be representative for all patients diagnosed with syndromic autism due to heterogeneity in clinical and behavioral profiles. Thus, replication of this study in other patients diagnosed with syndromic autism such as fragile X syndrome, * MECP2* duplication syndrome would be a valuable contribution to the literature. The study’s limitations also include proxy (parent/caregiver) perspectives regarding a child’s HRQoL. This implies that reported estimates might be impacted by bias due to proxy parental reporting of children’s HRQoL as the evidence shows that caregivers tend to underestimate the HRQoL of their children compared with a child’s self-report (Wolke, 2016; Verrips et al., 2001). COVID-19 questions had not been fully validated when the data was collected which is also a limitation. Furthermore, due to the nature of data collection we were not able to confirm the genetic diagnosis in each participant. Despite the data from our study sample deriving from socioeconomically diverse populations of patients the sample is not diverse in terms of race/ethnicity and family make-up. Thus, reported results might not be generalizable to more diverse samples. Finally, the empirical methods used may not fully control for selection effects caused by non-random participation in our data collection. Thus, the reported empirical relationships are not necessarily causal.

### Conclusions and Implications

The findings of this study have a number of implications for HRQoL and the living environments for children with syndromic autism, as well as, for autistic children more generally. The results of our study imply that caregiver access to mental health treatments must continue despite challenges imposed by an outbreak. This is particularly urgent given the recent evidence which shows that caregivers’ stress and anxiety levels might be directly linked to HRQoL outcomes in children (Spinelli et al., 2020; Ueda et al., 2021). Thus, it is imperative to design novel targeted interventions such as telehealth and integrated artificial intelligence systems aimed at ensuring that access to health services continues during potential future outbreaks.

Our study shows that time spent outdoors and time watching TV had opposite effects on HRQoL and on problematic behaviors in ASD children. Consequently, interventions incentivizing time outdoors and supporting play time outdoors might meaningfully improve HRQoL in children with syndromic autism. Understanding specific outdoor activities and their contribution to HRQoL among children with syndromic autism might be another fruitful area of investigation. Further studies conducted from multiple perspectives are needed to understand the mechanisms of HRQoL outcomes in children with syndromic autism. Our study demonstrates that HRQoL and behavioral outcomes reported earlier during the pandemic were generally worse compared to those reported later. While our models are not able to fully explain those results, these findings might suggest evidence of adaptive response due to psychological coping mechanisms which should be explored in future research.

Overall, our study implies that COVID-19-type measures aimed at stopping the transmissibility of infections will likely result in negative lifestyle changes associated with decrements in HRQoL and increase the likelihood of problematic behaviors in children with syndromic ASD. However, certain restrictions might increase activities which are likely to have a positive impact on children’s HRQoL and behaviors such as time spent outdoors. Strategies aimed at increasing time outdoors or time away from digital devices might protect ASD children from harmful consequences of restrictions to prevent the spread of respiratory infections.

## Appendix: COVID-19 Questions

See Table 9.

**Table 9:** COVID-19 Questions

*Questions*	
Since becoming aware of the COVID-19 outbreak, how often has your child: seemed in a daze	
Not at all	
Often	
Sometimes	
Since becoming aware of the COVID-19 outbreak, how often has your child: startled easily	
Not at all	
Often	
Sometimes	
Since becoming aware of the COVID-19 outbreak, how often has your child: had angry outburst	
Not at all	
Often	
Sometimes	
Since becoming aware of the COVID-19 outbreak, how often has your child: had difficulty sleeping	
Not at all	
Often	
Sometimes	
Does your child have access to a yard or other outdoor space where you live for outdoor play?	
Yes	
No	
On average, how much time has your child spent outdoors each day during the COVID-19 outbreak?	
1 hour or less	
1-2 hours	
2-3 hours	
>4 hours	
Rarely went out	
Since the COVID-19 outbreak began, on average, how many hours per day does your child sit and watch T.V. or videos? Please exclude school based activities.	
1 hour	
2 hours	
3 hours	
4 hours	
5 hours or more	
Less than 1 hour	
My child does not watch T.V. or videos	
Since the COVID-19 outbreak began, on average, how many hours per day does your child use a computer or play computer games, portable video games? Please exclude school based activities.	
1 hour	
2 hours	
3 hours	
4 hours	
5 hours or more	
Less than 1 hour	
My child does not have screen time on any devices	
Prefer not to answer	
On average, how much in-person, phone, or video contact has your child had with family/friends outside of the household during COVID-related school closures?	
Several times per day	
Don’t know	
Every few days	
Once per week	
Less than 1 hour	
Prefer not to answer	
In general, how successful has your child been in completing the recommended homeschool activities?	
Not at all successful	
Prefer not to answer	
Somewhat successful	
Very successful	

## Footnotes

1 Appendix A provides questions used to define these dependent variables.

2 WIC is a U.S. federal program that provides in-kind nutritional support for low-income pregnant and postpartum women and young children, and it serves approximately half of all infants, over a quarter of pregnant and postpartum women (Oliveira, 2009).

3 Furthermore, we tested the hypothesis that PMD patients before and after the cutoff date were different in terms of socio-demographic covariates. Thus, a linear probability model was designed to predict before or after cutoff date participation status as a function of child’s age, sex, caregiver’s race, family income, primary language spoken at home. However, the evidence from a linear probability model showed that the socio-demographic covariates were not significant predictors of before or after the cutoff date participation status.
